# miR-21 modulates the effect of EZH2 on the biological behavior of human lung cancer stem cells *in vitro*

**DOI:** 10.18632/oncotarget.20006

**Published:** 2017-08-07

**Authors:** Hui Xia, Wen Zhang, Baoshi Zhang, Yingnan Zhao, Yunlong Zhao, Shaojun Li, Yang Liu

**Affiliations:** ^1^ Department of Thoracic-Cardio Surgery, First Affiliated Hospital of PLA General Hospital, Beijing, China; ^2^ Department of Thoracic Surgery, PLA General Hospital, Beijing, China; ^3^ Medical School of PLA, Beijing, China

**Keywords:** miR-21, EZH2, lung cancer stem cells, radiotherapy, chemotherapy

## Abstract

Non-small-cell lung cancer has a high mortality rate and poor prognosis. Therefore, novel therapeutic approaches are urgently needed to enhance patient survival rates. In this study, we investigated the effects of miR-21 and EZH2 on the biological behavior of human lung cancer stem cells *in vitro*. We found increased expression of EZH2 and miR-21 in LCSCs, and miR-21 overexpression increased EZH2 levels in LCSCs. In addition, EZH2 and miR-21 knockdown increased the sensitivity of LCSCs to chemo- and radiation therapy, and exogenous EZH2 expression rescued the effects of anti-miR-21. Cell proliferation was reduced by 39.2% and 69.7% in the presence of radio*-* or chemotherapy combined with anti-miR-21 transfection, respectively. The downstream molecules included Cdc2, cyclin B1, and Bcl-2, which are involved in the regulation of cell cycle and apoptosis and which could themselves be reduced or enhanced by changes in miR-21 and EZH2 levels in LCSCs. This study demonstrates the direct relationship between miR-21 and EZH2 which was increased by 43% after the application of the miR-21 mimic. Above data indicates that these two molecules can influence the biological behavior of LCSCs by altering their corresponding targets. Our findings support the potential roles of miR-21 and EZH2 in improving the therapeutic efficacy of clinical lung cancer treatments.

## INTRODUCTION

Lung cancer, the leading cause of cancer-related mortality worldwide, has a high mortality rate and poor prognosis—particularly non-small-cell lung cancer (NSCLC), which accounts for approximately 80% of all lung cancers and has a 5-year overall survival rate of < 15% [1, 2]. In addition, approximately 40% of all NSCLC patients present with unresectable stage III disease or inoperable disease [3, 4].

Despite recent advances and developments in therapeutic strategies, including surgery, chemotherapy, and radiotherapy, poor survival remains an issue due to the high systemic toxicity and drug resistance that limit successful outcomes in most cases [5, 6]. Therefore, the development of novel approaches for the diagnosis, treatment, and prevention of NSCLC, including targeted gene therapy as an adjuvant modality or as a radiosensitizer, is urgently needed to enhance patient survival.

Cancer stem cells, which exhibit stem cell-like self-renewal capabilities, are involved in the initiation and development of cancers such as breast [7], pancreatic [8], brain [9] and lung cancers [10]. Recent studies [11, 12] and our previous report [13] supported the role of side population (SP) cells, which can be isolated from human lung cancer cell lines and exhibit significant tumor-initiating activity and characteristic proteins, such as CD133, ABCG2 and multi-drug resistance transporters. Lung cancer stem cells (LCSCs) represent a novel target for clinical cancer therapy, but the underlying mechanisms for LCSC tumorigenesis remain unknown and is an urgent area of study.

Recently, significant advances in our biological and molecular understanding of cancer have facilitated the development of agents targeted to specific molecules in the treatment of NSCLC [14, 15]. Novel anti-lung cancer agents that target proteins such as EGFR or *Enhancer of zeste homolog 2* (EZH2) combined with chemotherapy or radiotherapy have been reported [16, 17]. EZH2, a catalytically active component of the PRC2 complex, is one of the targets currently being evaluated for the treatment of lung cancer. Various studies have identified that abnormal expression of EZH2, a potential marker for distinguishing aggressive from indolent or benign cancers, contributes to the tumorigenesis of several malignancies, including melanoma, prostate, breast, bladder, and endometrial cancers, and results in proliferative advantages for eukaryotic cells by affecting the key pathways that control cellular growth arrest and differentiation [18, 19]. As a transcriptional repressor, EZH2 controls cellular growth and proliferation by promoting S-phase entry and the G_2_/M transition [20, 21]. EZH2 also promotes the repression of specific genes, a process that also involves histone deacetylation by histone deacetylase-1 (HDAC-1), which interacts with EZH2 via its PRC2-binding partner EED [22, 23].

microRNAs (miRNAs) are a class of short noncoding RNAs that have been demonstrated to regulate the expression of genes governing tumorigenic processes by targeting mRNAs for degradation or translational inhibition. miRNAs play key roles in lung cancer development, including cellular differentiation, apoptosis, invasion and the cell cycle [24-26]. miR-21 is overexpressed in several human malignancies, including NSCLC. miR-21 expression in lung cancer can be considered a biomarker for poor prognosis, chemotherapeutic response and radioresistance [27-29]. miR-21 has been demonstrated to play a important role in the radioresistance of cancers, including glioblastoma, breast cancer, rectal cancer. The inhibition of miR-21 expression sensitizes cancer cells to topotecan and gemcitabine [30-31]. miR-21 can modulate the histone deacetylase (HDAC) expression and Akt/Gsk3β pathway [32]. Our recent study also demonstrated that the down regulation of miRNA-21 sensitizes radioresistant NSCLC A549 cells to IR by inhibiting the PI3K/Akt signaling pathway [33]. In addition, effect of EZH2 mediated epigenetic gene silencing is dependent on HDAC activity [34-35]. And our data also reported that EZH2 regulate cell cycle through its SET-domain regulated H3K27me3 activity via p53/p21 downstream pathway [36].

Few studies have reported on the function of miRNAs, particularly miR-21, in LCSCs. Thus, in this study, we tested the hypothesis that down regulation of miR-21 and EZH2 expression level *via* anti-miR-21 or EZH2 shRNA reduce LCSC growth, thereby altering lung cancer development and progression. The underlying mechanism and the related pathway involving miR-21 and EZH2, which are important biomarkers and target molecules in the clinical treatment for lung cancer, were explored. Our results provide direct evidence for the application of miR-21 or EZH2 knockdown in future clinical treatment strategies for NSCLC patients.

## RESULTS

### EZH2 expression in lung cancer stem cells

**Figure 1:**
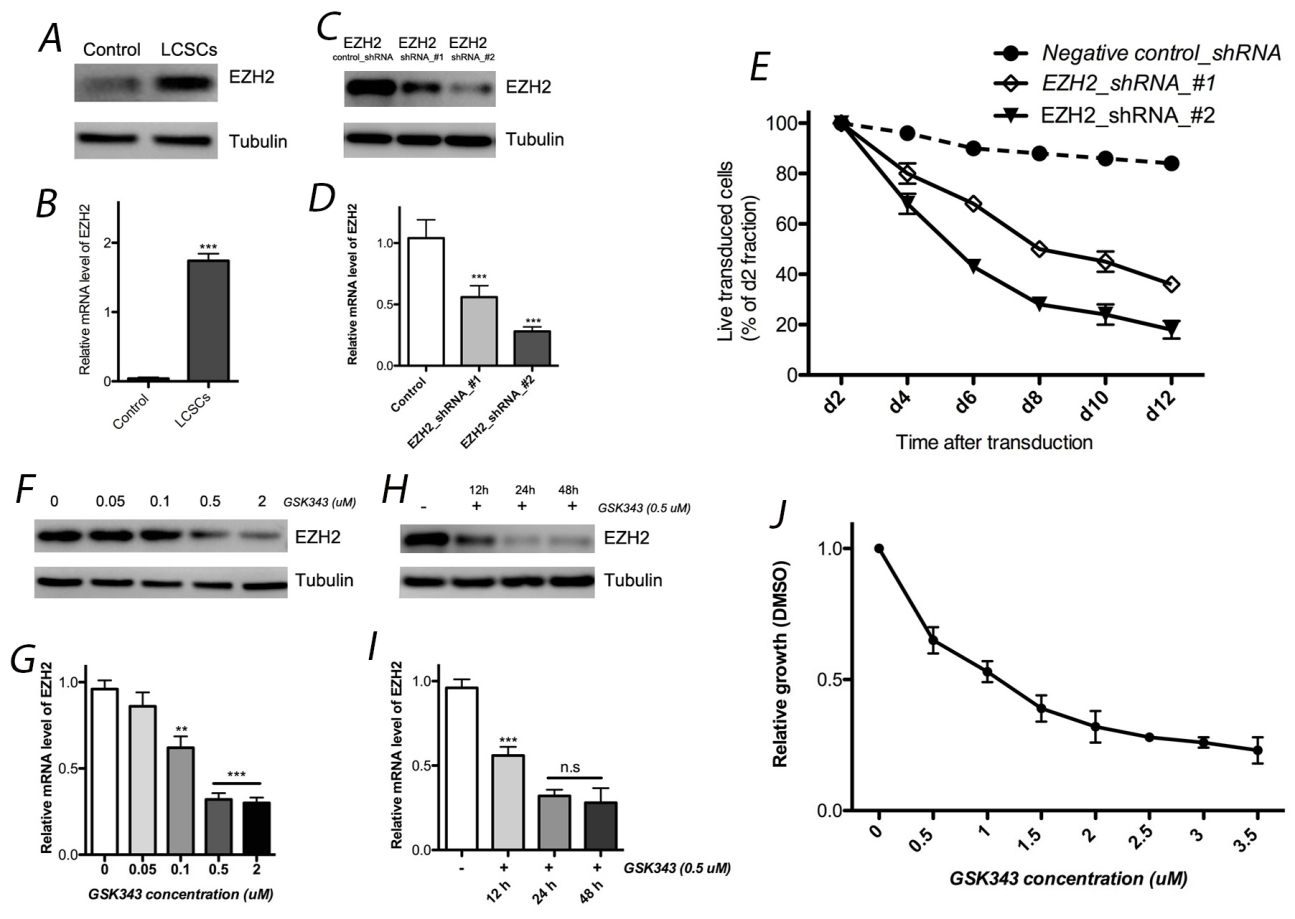
EZH2 expression in LCSCs EZH2 expression in LCSCs by western blotting **(A)** and real-time quantitative RT-PCR **(B)** analyses. Both EZH2 shRNAs significantly decreased EZH2 expression in LCSCs at the protein **(C)** and mRNA **(D)** levels; **(E)** effects of EZH2_shRNA on cell growth of LCSCs; EZH2 protein **(F)** and mRNA **(G)** expression was affected by different concentration of GSK343; effect of GSK343 with different incubation time were also observed by western blotting **(H)** and real-time quantitative RT-PCR **(I)** analyses; **(J)** cell viability was evaluated after 4 days of incubation with GSK343. Each experiment was performed independently at least 3 times. Each experiment was performed in triplicate, ^***^P < 0.001, ^**^P < 0.01, ^*^P < 0.05.

To detect EZH2 in LCSCs, we performed real-time quantitative RT-PCR and western blotting analyses. Both analyses revealed high levels of EZH2 in LCSCs (Figure 1, Supplementary Figure 1). These results were consistent with previous reports [37, 38], which previously indicated a relationship between EZH2 expression and lung cancer development.

Two independent shRNAs were used to knockdown EZH2 to assess its functional significance in LCSC. Both EZH2 shRNAs significantly decreased EZH2 expression at the protein and mRNA levels. In addition, we observed toxicity in LCSCs after the transfection of these two EZH shRNAs. The GFP- positive populations of live cells were normalized to that of the control groups (negative shRNA) first and to the day-2 fraction (Figure 1). Each experiment was performed independently at least 3 times.

We used the specific pharmacological inhibitor GSK343 to down regulate expression level of H3K27me3 and EZH2 activity. Next, cell viability was evaluated after 4 days of incubation with GSK343. We observed that the effect of GSK343 on cell growth was expressed in dose-dependent manner, and 1 μM GSK343 reduced cell viability by approximately 50% (Figure 1). These data indicate that the pharmacological inhibitor can mimick EZH2 knock down which affects LCSC survival and represents a potential therapeutic modality for lung cancer patients.

### Effect of miR-21 on EZH2 expression

**Figure 2:**
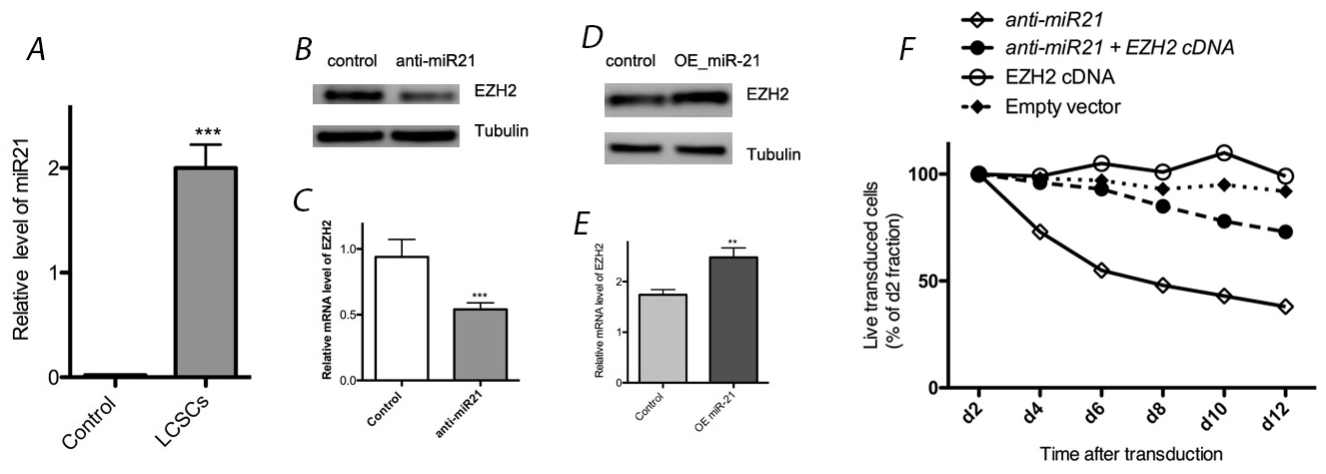
Effect of miR-21 on EZH2 expression in LCSCs **(A)** Relative expression of miR-21 was detected in LCSC by real-time quantitative RT-PCR; **(B)** & **(C)** EZH2 expression was reduced by transfection with anti-miR-21 in LCSC; **(D)** & **(E)** EZH2 expression was enhanced by transfection with OE miR-21 in LCSC; **(F)** transfection of anti-miR-21 can induce toxicity in LCSCs, and exogenous EZH2 expression rescued LCSCs from anti-miR-21-induced toxicity. Each experiment was performed in triplicate, ^***^P < 0.001, ^**^P < 0.01, ^*^P < 0.05.

We observed high expression levels of miR-21 in LCSC by real-time quantitative RT-PCR, as well as its down regulation after transfection with anti-miR-21 compared with controls (Figure 2). Our western blotting results suggested that EZH2 expression was down regulated by the application of anti-miR-21, as the levels decreased by 58% compared with controls. Transfection of anti-miR-21 can induce toxicity in LCSCs (Figure 2). To determine whether the effects of miR-21 down regulation on cell proliferation are related to the expression of EZH2, a rescue experiment was performed to observe the effects of EZH2 up regulation. Indeed, exogenous EZH2 expression rescued LCSCs from anti-miR-21-induced toxicity (Figure 2). Furthermore, miR-21 over expression through transfection with a miR-21 mimic (OE miR-21) also increased EZH2 expression. Compared with controls, EZH2 expression was increased by 43% after the application of the miR-21 mimic (Figure 2). Based on theses results, miR-21 appears to enhance EZH2 expression in LCSCs.

### Colony-formation assays

**Figure 3:**
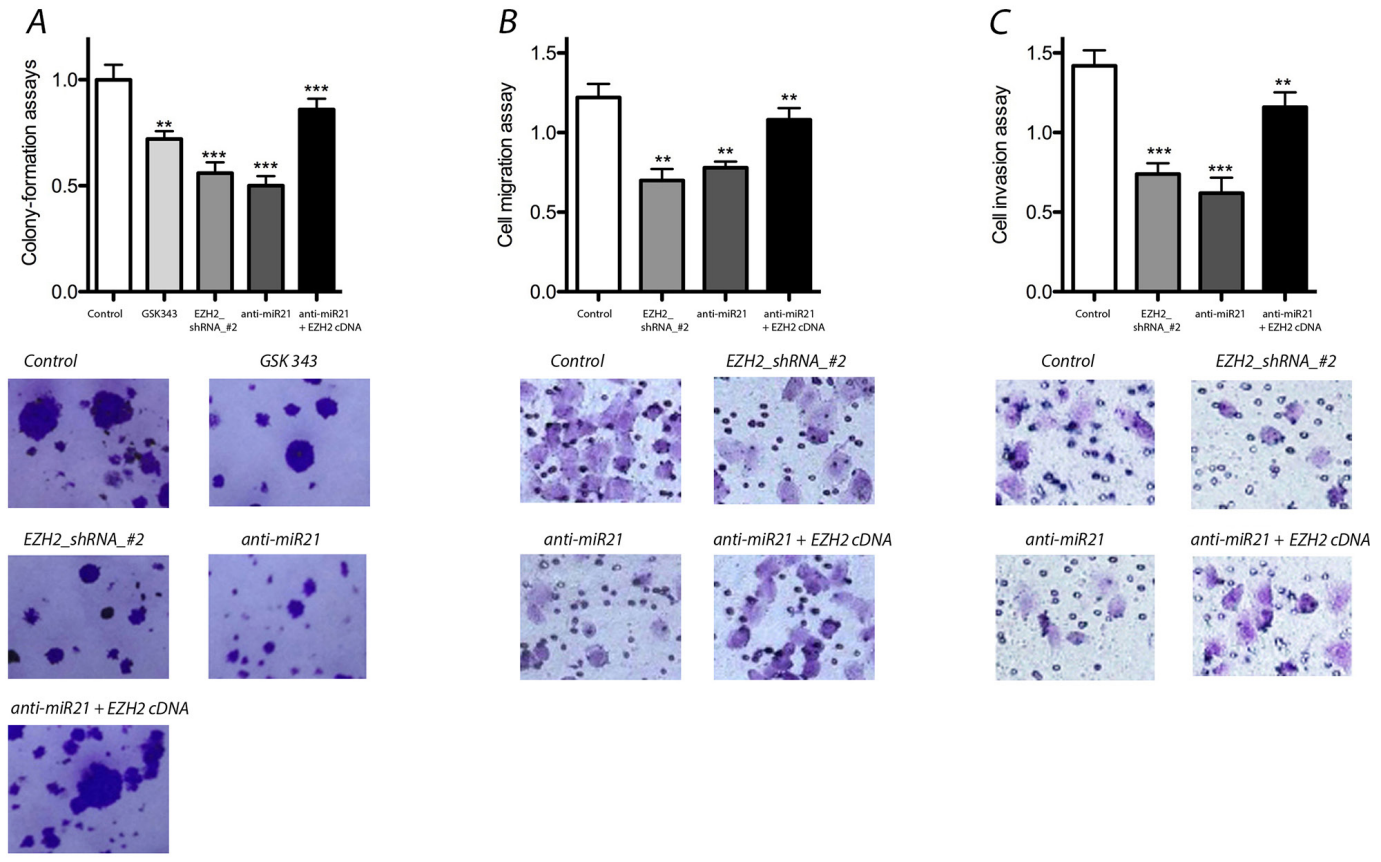
Effect of miR-21 and EZH2 on colony-formation, migration and invasion of LCSCs **(A)** Colony-formation assays was decreased significantly after EZH2 or miR-21 knockdown, but rescued the effects of anti-miR-21 after overexpression of exogenous EZH2 in LCSCs; **(B)** & **(C)** cell migration and invasion were reduced significantly by EZH2 or miR-21 knock down, but exogenous EZH2 over expression rescued the effects of anti-miR-21 in LCSCs. Each experiment was performed in triplicate, ^***^P < 0.001, ^**^P < 0.01, ^*^P < 0.05.

We performed colony-formation assays to assess the effects of EZH2 knockdown with shRNA or anti-miR-21 treatment. Both EZH2 and miR-21 knockdown significantly reduced colony formation. GSK343 elicited similar effects. In addition, we performed an EZH2 cDNA mutant experiment to rescue the effect of anti-miR-21 treatment. Our results indicated that the colony-formation capacity that was reduced by anti-miR-21 could be rescued by the over expression of exogenous EZH2 (Figure 3).

### Migration and invasion assays

We evaluated the effects of miR-21 and EZH2 on cell migration and invasion by down or up regulating miR-21 and EZH2 expression. Compared with controls, the suppression of either miR-21 or EZH2 resulted in significantly decreased well migration and invasion activity. Similarly, exogenous EZH2 over expression rescued the effects of anti-miR-21 in LCSCs (Figure 3). Thus, these data demonstrate that both miR-21 and EZH2 are important for cell migration and invasion.

### Effects on cell proliferation

**Figure 4:**
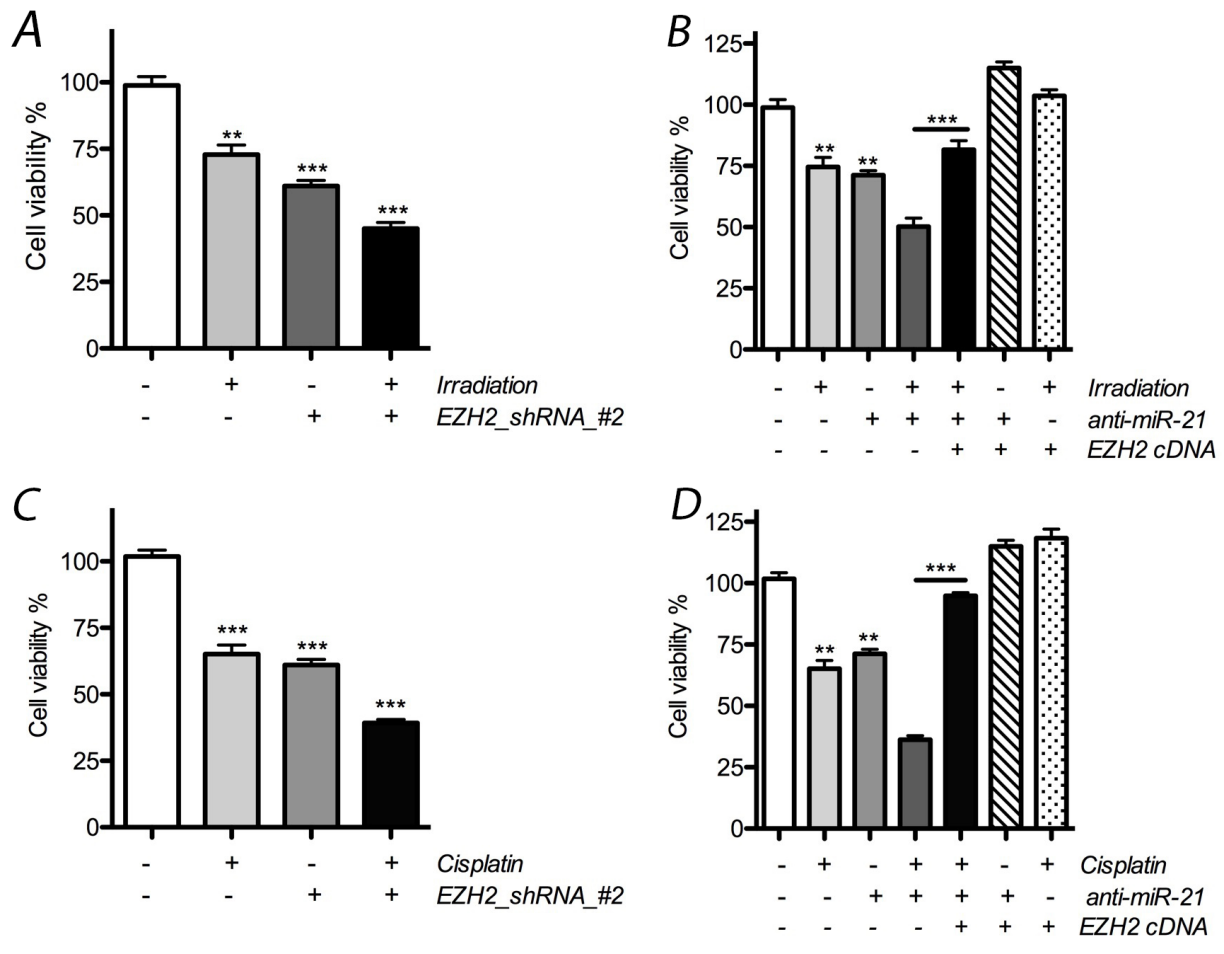
Effect of miR-21 or EZH2 on cell proliferation of LCSCs **(A)** Combinative effect on cell proliferation was obtained obviously after treated with radiotherapy and EZH2_shRNA; **(B)** cell proliferation was reduced after combined with radiotherapy and anti-miR-21, but rescued above effects after overexpression of exogenous EZH2 in LCSCs; **(C)** combinative effect on cell proliferation was obtained obviously after treated with cisplatin and EZH2_shRNA; **(D)** cell proliferation was reduced after combined with cisplatin and anti-miR-21, but rescued above effects after ectopic expression of EZH2 in LCSCs. Each experiment was performed in triplicate, ^***^P < 0.001, ^**^P < 0.01, ^*^P < 0.05.

In the following experiments, we tested the hypothesis that changes in miR-21 or EZH2 expression influence the effects of radio*-* or chemotherapy on LCSC proliferation. After treatment with radiotherapy alone, the number of LCSCs was reduced by 28.5%, and a significant effect was observed in the presence of EZH2 shRNA, where it was reduced by at least 51.3% (Figure 4). In addition, our results indicate that the reduction of miR-21 by anti-miR-21 transfection also resulted in additive anti-proliferative effects on cells when combined with radiation therapy, where the number of LCSCs was reduced by 39.2% and 26.3% in the presence or absence, respectively, of anti-miR-21 transfection.

In cisplatin-treated cells, the number of cells was reduced by at least 29.7%, and an additive effect was observed in the presence of EZH2 shRNA, where cell numbers were reduced by 55.1%. When combined with anti-miR-21 transfection, the number of cells was further reduced by 69.7% after chemotherapy and 31.2% for the group that received cisplatin alone. Thus, our data demonstrate that EZH2 shRNA or anti-miR-21 can elicit additive anti-proliferative effects when combined with chemo- or radiotherapy on LCSCs. Interestingly, the rescue effect of anti-miR-21 was also observed after the ectopic expression of EZH2 (Figure 4).

### Effects on cell cycle

We investigated the cell cycle alterations induced by the altered expression of miR-21 or EZH2 in LCSCs by FACS. We also evaluated the combinative effects of chemo- or radiotherapy on cell cycle progression after altering the expression levels of miR-21 or EZH2 by transfection. Our results showed that the S-phase fraction of cells decreased by 13% or 22% in the presence of anti-miR-21 or EZH2 shRNA, respectively. Upon cisplatin treatment, the S-phase fraction of cells decreased by 11%, but it decreased more significantly in the presence of anti-miR-21 or EZH2 shRNA (by 26% or 29%, respectively). In addition, the number of cells in G_2_/M phase increased by 21% or 19% after transfection with anti-miR-21 or EZH2 shRNA, respectively, and it increased more significantly upon chemotherapeutic treatment by 32% or 35% in the presence of anti-miR21 or EZH2 shRNA, respectively (Supplementary Table 1).

Similar effects were observed upon radiotherapy, where additive changes were also observed. The S-phase fraction of cells decreased by 20% and 26% in the presence of anti-miR-21 or EZH2 shRNA, respectively, after combined treatment, whereas it only decreased by 13% after radiotherapy treatment. In contrast, the number of cells in G_2_/M phase increased by 41% or 38% after above combinatorial therapy but only resulted in a 23% increase after radiotherapy. Further experiments were also performed to observe the rescue effect of EZH2 up regulation (Supplementary Table 2).

**Figure 5:**
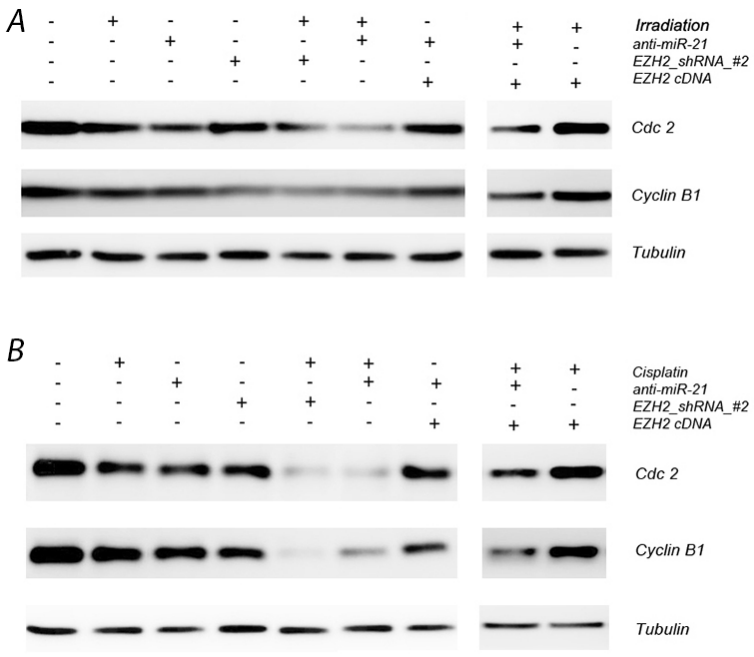
Effect of miR-21 or EZH2 on cell cell-cycle-related biomarkers in LCSCs **(A)** Cdc2 and cyclin B1 expression after transfection with anti-miR-21 or EZH2 shRNA in the presence or absence of irradiation treatment on LCSCs; **(B)** Cdc2 and cyclin B1 expression after transfection with anti-miR-21 or EZH2 shRNA in the presence or absence of cisplatin treatment on LCSCs. Each experiment was performed in triplicate.

We next detected the expression levels of cell-cycle-related biomarkers including cell division cycle 2 (Cdc2) and cyclin B1 after transfection with anti-miR-21 or EZH2 shRNA by western blotting. Cdc2 and cyclin B1 decreased markedly both in presence of either anti-miR-21 or EZH2 shRNA. After combined treatment with radio*-* or chemotherapy, Cdc2 and cyclin B1 also changed significantly (Figure 5, Supplementary Figure 2). Our results demonstrate that down regulation of Cdc2, cyclin B1 expression correlates with altered cell cycle dynamics after changes in miR-21 or EZH2 levels, which might help to improve the therapeutic effects of chemotherapy or radiation therapy on LCSCs.

### Effects on apoptosis

To evaluate the effects of miR-21 or EZH2 on LCSC apoptosis, we assessed apoptotic rates by FACS. The percentages of apoptotic cells were 11.2 ± 0.3% and 12.1 ± 1.1% in the presence of anti-miR-21 or EZH2 shRNA, respectively. In the single chemo- or radiation therapy groups, the percentages were 14.1± 0.5% and 14.9 ± 0.2%, respectively. In addition, additive effects on cell apoptosis were also observed after combined treatment, where the percentages were 18.5 ± 0.3% and 21.9 ± 0.9% after irradiation coupled with anti-miR-21 or EZH2 shRNA, respectively, and 21.3% ± 0.2 and 22.2 ± 0.4% for chemotherapy coupled with anti-miR-21 or EZH2 shRNA, respectively (Supplementary Tables 3 and 4).

**Figure 6:**
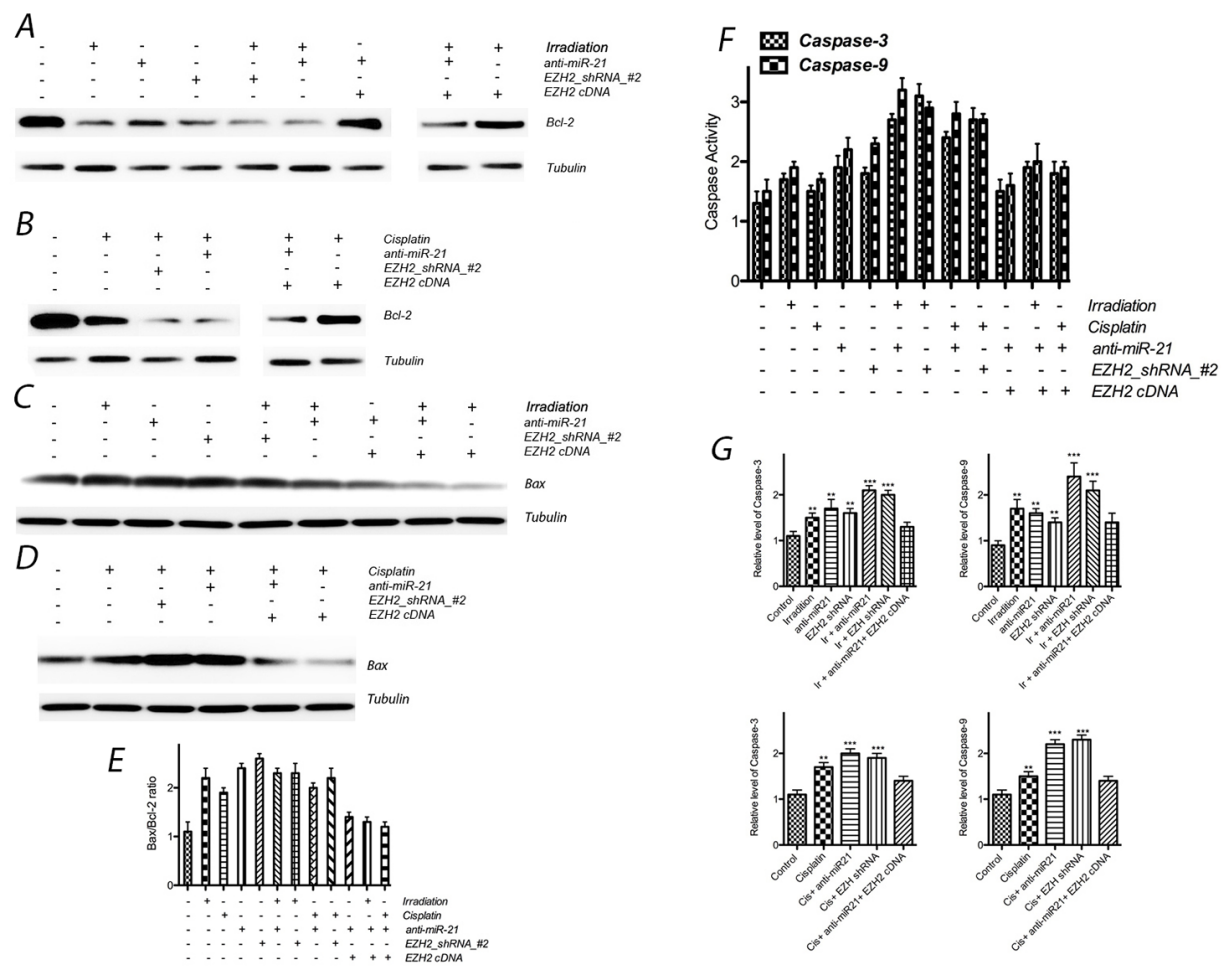
Effect of miR-21 or EZH2 on cell apoptosis of LCSCs **(A)** Bcl-2 expression after transfection with anti-miR-21 or EZH2 shRNA in the presence or absence of irradiation treatment on LCSCs; **(B)** Bcl-2 expression after transfection with anti-miR-21 or EZH2 shRNA in the presence or absence of cisplatin treatment on LCSCs; **(C)** Bax expression after transfection with anti-miR-21 or EZH2 shRNA in the presence or absence of irradiation treatment on LCSCs; **(D)** Bax expression after transfection with anti-miR-21 or EZH2 shRNA in the presence or absence of cisplatin treatment on LCSCs; **(E)** the ratio between Bax and Bcl-2 also increased obviously after above treatment; **(F)** caspase activity were observed after above combinative treatment; **(G)** caspase exression were observed after above combinative treatment. Each experiment was performed in triplicate, ^***^P < 0.001, ^**^P < 0.01, *P < 0.05.

Furthermore, to assess the expression of apoptosis-related molecular markers induced by the above treatments, we evaluated Bcl-2 and Bax expression by Western blotting. In addition, we assessed caspase-3 and caspase-9 activities using an assay kit. After transfection of either anti-miR-21 or EZH2 siRNA, Bcl-2 expression markedly decreased and Bax expression increased, and thus the ratio between Bax and Bcl-2 also increased obviously after above treatment; caspase-3 and caspase-9 activities were increased significantly, in addition, expression of caspase-3 and caspase-9 were also enhanced after knock down miR-21 or EZH2. The expression of the above apoptosis-related molecular markers significantly decreased in the combined treatment group compared with those in the single-treatment groups. In addition, the exogenous expression of EZH2 also elicited a rescue effect against anti-miR21-induced apoptosis (Figure 6).

## DISCUSSION

Our data provide the following novel findings: (1) increased expression of EZH2 and miR-21 is observed in LCSCs; (2) miR-21 overexpression increases EZH2 levels in LCSCs; (3) knock down of EZH2 and miR-21 sensitizes LCSCs to chemo- and radiation therapy; (4) exogenous expression of EZH2 rescues the effects of anti-miR-21; (5) the downstream effectors, including Cdc2, cyclin B1, Bax and Bcl-2, which are involved in cell cycle progression and apoptosis, are up or down regulated by alterations in miR-21 and EZH2 levels in LCSCs. Thus, our data demonstrate the direct relationship between miR-21 and EZH2, which helps to explain the fact that these two molecules can enhance the biological behavior of LCSC through the modification of their targets. Such effects suggest the potential for targeting miR-21 and EZH2 to improve the therapeutic efficacy of lung cancer treatments.

Combining radio- or chemotherapy with biological agents for targeting cancer has been investigated to improve therapeutic outcomes and decrease toxicity to normal tissues due to specific tumor response, effects that were observed in our study. However, resistance to radio- or chemotherapy, which impairs therapeutic efficacy, has been reported as significant reasons for the poor long-term survival for most NSCLS patients [39, 40]. Thus, investigations of new methods and therapeutic targeting, which can reduce resistance and increase therapeutic efficacy, have recently garnered increased attention. Our previous studies also demonstrated that the knock down EZH2 and miR-21 can sensitize radioresistant NSCLC A549 cells [33, 36]. In the current study, the purpose was to test the hypothesis that the down regulation of EZH2 and miR-21 can have additive effects when combined with radiation or chemotherapy in LCSCs.

It has been reported that EZH2 is an oncogene involved in tumor progression, and miR-21 is also a biomarker for the tumorigenic processes, particularly for the pathological development of lung cancer [41, 42]. However, few studies have focused on these two molecules in LCSC. Our data provide positive evidence that EZH2 and miR-21 are highly expressed in LCSCs, which are suggestive of their important roles in the biological behavior of lung cancer.

In addition, few studies have focused on the up regulation of EZH2 and its underlying mechanisms. Thus, in this study, we experimentally evaluated the relationship between EZH2 and miR-21. Taken together, our results indicate that both miR-21 and EZH2 are essential for the proliferative potential and colony-formation capacity of lung cancer cells. The expression of EZH2, which can rescue the effects of anti-miR-21, could also be a down stream effector of miR-21.

EZH2 knockdown can increase the expression of p53 and p21, which can significantly modulate the levels of Cdc2 and cyclin B1 in both A549 and HTB-56 cells [36]. However, whether EZH2 can also elicit similar effects in LCSCs is unknown. Our results indicate that the down regulation of EZH2 or miR-21 can regulate cell cycle distribution, arresting cells in G_2_/M and delaying cell cycle progression. In addition, cell cycle dynamics were more significantly altered in the combination treatment groups compared with single treatment groups. The effects of miR-21 on cell cycle were rescued by the exogenous over expression of EZH2.

In conclusion, our study provides direct evidence that miR-21 and EZH2 knockdown can reduce the biological behavior of human lung cancer stem cells *in vitro*. Our data indicate that the altered behavior is associated with biomarkers of cell cycle or apoptosis. In addition, EZH2 might represent a down stream effector of miR-21. Our findings can potentially be used to develop new therapeutic targets and treatments for NSCLC.

## MATERIALS AND METHODS

### Cell culture

The human lung adenocarcinoma A549 cell line was obtained from the American Type Culture Collection (*ATCC, Manassas, VA*), SP cells (*LCSCs*) were prepared as previously reported [11-13] and maintained in Dulbecco’s modified Eagle’s medium (DMEM, *Gibco, Carlsbad, CA, USA*) supplemented with 10% heat-inactivated FBS and 1% penicillin/ streptomycin at 37°C in a humidified incubator with 95% air and 5% CO_2_. The serum free DMEM/F12 including EGF (20 ng/mL; Pepro Tech), bFGF (20 ng/mL; PeproTech) and IGF1 (20 ng/mL; PeproTech) was used for SP cell culture. All procedures had the approval of the Ethics Committee of the PLA General Hospital.

### Transfection

Using Lipofectamine 2000 transfection reag-ent (*Invitrogen, Carlsbad, CA, USA*), cells were transfected with anti-miR-21 (*5’-UCAACAUCA-GUCUGA UAAGCUA-3’*) or the negative control (*NC, 5’-CAGUACUUUUG-UGUAGU ACAA-3’*), which were obtained from Ambion Inc. (*Austin, TX, USA*). In addition, miR-21 overexpression was achieved by transfecting cells with a synthetic miRIDIAN mimic or a negative control. Another group of cells was transfected with validated shRNA for EZH2 or a negative control vector (*Qiagen, Lafayette, CO, USA*) at a concentration of 100 nM using the Lipofectamine 2000 transfection reagent. The EZH2 gene targeting sequences were as follows: EZH2 sense, *TTCATGCAACACCCAACACT*; and EZH2 antisense, *GAGAGCAGCAGCAAACTCCT*. Furthermore, EZH2-mutant *(NM_ 152998, Origene, Beijing, China)* expression constructs were generated by cloning the corresponding cDNAs into the vector. At 6 h post-transfection, the medium was replaced with standard culture medium. After shRNA transfection, flow cytometry was performed to determine the toxicity of the shRNAs by quantifying the initial GFP-positive proportion of live cells. Doxycycline was used to induce the expression of the shRNA or exogenous cDNA expression.

### Western blotting

Western blotting was performed as following protocol. Cells were lysed in buffer containing 0.01 M Tris-HCl (pH 7.5), 150 mM NaCl, 0.01 M EDTA, 0.01 M EGTA, 1% Triton X-100, 0.01 M β-glycerophosphate, 0.01 M Na_3_VO_4_, and 1 μg/ml leupeptin supplemented with proteinase (*Sigma-Aldrich, St. Louis, MO, USA*) and phosphatase (*Sigma-Aldrich, St. Louis, MO, USA*) inhibitor cocktails. Protein concentrations of the cell lysates were determined using a BCA protein assay kit (*Pierce, Rockford, IL, USA*). All antibodies used in this study were obtained from Cell Signaling Technology *(Beverly, MA, USA)*, and β-actin (*Sigma-Aldrich, St. Louis, MO, USA*) was used to correct for potential unequal loading and transfer of proteins.

### Quantitative PCR

The mRNA expression levels of miR-21 and EZH2 were determined by quantitative PCR using predesigned assays (*Applied Biosystems,CA,USA*).

### Colony-formation assay

For the colony-formation assays, cells were seeded in triplicate into 6-well tissue culture plates and cultured for 12 days. Then, Giemsa staining was used to evaluate the number of colonies by light microscopy.

### Migration assay

For the transwell migration assays, cells were seeded into the upper chamber in serum-free medium. The lower chamber contained growth medium with 10% FBS and 100 ng/ml EGF (*Invitrogen, USA*). After 24 h, the cells that had migrated to the lower chamber were fixed with 1% paraformaldehyde and stained with 1% crystal violet. Cell migration was quantified using light microscopy.

### Invasion assays

Invasion was evaluated using the transwell system after transfection. Cells were seeded in the upper chamber that contained growth media with 10% FBS and 100 ng/ml EGF (*Invitrogen, USA*) for 24 h. Next, cells that passed through the filter were fixed with 1% paraformaldehyde and stained with hematoxylin. The number of invaded cells was quantified using light microscopy.

### Radiation treatment and chemotherapy

After the cells were transfected with anti-miR-21 or EZH2 shRNA, the cells in the radiation-treated group were γ-irradiated at a single dose of 2 Gy/min every 3 days for 2 weeks, and the cells in the chemotherapy group were treated with 30 nM cisplatin every 3 days for 2 weeks.

### Cell proliferation

Cell proliferation was evaluated using an MTT kit (*Roche Diagnostics, Germany*) after radio- or chemotherapy in the presence or absence of the transfections described above. Cells were seeded at a density of 5 × 10^3^ cells/well and were treated as described above. The cells in each treatment group were harvested by trypsinization at different time points, and the results were recorded using a Universal Microplate Spectrophotometer (*BioTek, VT, USA*). Experiments were performed in triplicate.

### Cell cycle analysis

To evaluate the cell cycle phase distribution, cells were seeded at a density of 1 × 10^5^ cells/well in six-well plates. As described in the protocol above, the cells were transfected and treated with radio- or chemotherapy. Next, the cells were harvested, washed and fixed with 70% (v/v) ethanol overnight at 4°C. RNase A (100 µg/ml) and propidium iodide (PI, 50 mg/ml) were incubated with cells for 30 min. The cell cycle phase distribution was assessed by flow cytometry (*Becton Dickinson, San Jose, CA, USA*). The experiments were performed in triplicate. Primary antibodies against Cdc2 and cyclin B1 (*1:1,000; Cell Signaling Technology, Beverly, MA, USA*) were used to assess protein expression by western blotting.

### Cell apoptosis analysis

To analyze apoptosis, cells were seeded at a density of 1 × 10^5^ cells/well in six-well plates. As detailed above, the cells were transfected and treated with radio- or chemotherapy. The cells were incubated with PI and annexin V-FITC to assess apoptosis by flow cytometry (*Becton Dickinson, San Jose, CA, USA*). The primary antibody against Bax and Bcl-2 (*1:1000; Santa Cruz Biotechnology, CA, USA*) was used to assess its expression by western blotting. Caspase activities was also observed by the specific assay kit.

## SUPPLEMENTARY MATERIALS FIGURES AND TABLES


