# Supplementary material for: miR-21 modulates the effect of EZH2 on the biological behavior of human lung cancer stem cells in vitro
Source: Oncotarget. 2017 Aug 7;8(49):85442–51. doi: 10.18632/oncotarget.20006 (PMC5689621; doi:10.18632/oncotarget.20006)
Supplement: Supplementary file 1 [file oncotarget-08-85442-s001.pdf]

miR-21 modulates the effect of EZH2 on the biological behavior of human lung cancer stem cells *in vitro*

SUPPLEMENTARY MATERIALS

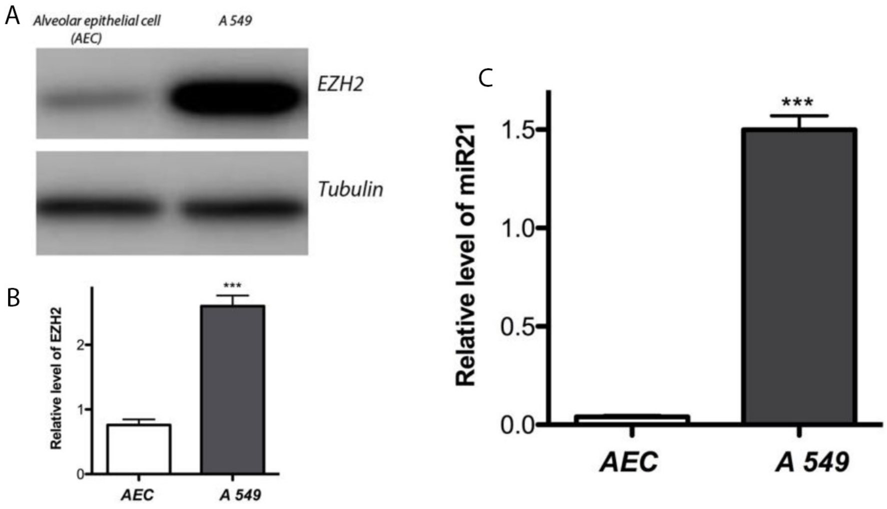

Supplementary Figure 1: Expression of EZH2 and miR21 both in AEC and A549 cell. EZH2 protein **(A)** and mRNA **(B)** expression were observed both in Alveolar epithelial cell (AEC) and A549 cell (control). miR21 (**C**) level was also detected in above cells. Low expression of EZH2 in alveolar epithelial cell compared with A549 cell, and miR21 level was not observed in AEC, but high level in A549 cell. Each experiment was performed in triplicate, ∗∗∗P < 0.001, ∗∗P < 0.01, ∗P < 0.05.

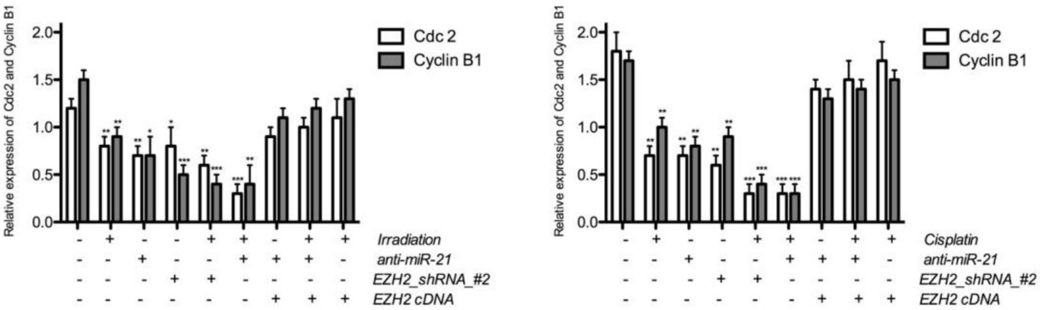

Supplementary Figure 2: Relative expression of Cdc 2 and Cyclin B1 after treatment. (**A**) Relative expression of Cdc 2 and Cyclin B1 were observed after irradiation treatment combined with anti-miR-21, EZH2 shRNA or cDNA transfection in LCSCs respecitvely. **(B)** Relative expression of Cdc 2 and Cyclin B1 were observed after Cisplatin treatment combined with anti-miR-21, EZH2 shRNA or cDNA transfection in LCSCs respecitvely. Each experiment was performed in triplicate, ∗∗∗P < 0.001, ∗∗P < 0.01, ∗P < 0.05.

Supplementary Table 1: Effect of miR-21 or EZH2 on cell cycle of LCSCs after Irradiation treatment ($\bar{x} \pm s$, n = 6)

Group	G1 (%)	S (%)	G2/M (%)
Control	73.5±0.2	8.5±0.4	18.0±0.4
Irradiation	63.5±0.1	13.2±0.4	23.3±0.1
Anti-miR-21	66.5±0.3	13.1±0.1	21.4±0.2
EZH2_shRNA_#2	59.5±0.3	22.4±0.2	19.1±0.5
Irradiation + Anti-miR-21	48.5±0.1	20.0±0.2	41.5±0.3
Irradiation + EZH2_shRNA_#2	65.1±0.3	26.5±0.2	38.4±0.6
Irradiation + EZH2 cDNA	74.1±0.1	9.2±0.3	16.7±0.6
Anti-miR-21 + EZH2 cDNA	70.0±0.5	11.5±0.6	19.5±0.2
Irradiation + Anti-miR-21 + EZH2 cDNA	64.5±0.1	15.4±0.3	21.1±0.2

Supplementary Table 2: Effect of miR-21 or EZH2 on cell cycle of LCSCs after Cisplatin treatment ($\bar{x} \pm s$, n = 6)

Group	G1 (%)	S (%)	G2/M (%)
Control	73.5±0.2	8.5±0.4	18.0±0.4
Cisplatin	64.2±0.1	11.3±0.5	25.5±0.2
Anti-miR-21	66.7±0.3	13.1±0.1	21.2±0.6
EZH2_shRNA_#2	59.5±0.2	22.4±0.2	19.1±0.3
Cisplatin + Anti-miR-21	42.1±0.1	26.7±0.4	32.2±0.6
Cisplatin + EZH2_shRNA_#2	36.4±0.3	29.1±0.1	35.5±0.5
Cisplatin + EZH2 cDNA	72.5±0.1	14.2±0.3	14.3±0.2
Anti-miR-21 + EZH2 cDNA	70.0±0.5	11.5±0.6	19.5±0.2
Cisplatin + Anti-miR-21 + EZH2 cDNA	60.5±0.1	17.2±0.2	23.3±0.6

Supplementary Table 3: Effect of miR-21 or EZH2 on cell apoptosis of LCSCs after Irradiation treatment ($\bar{x} \pm s$, n = 6)

Group	Apoptosis (%)
Control	2.9±0. 4
Irradiation	14.9 ± 0.2
Anti-miR-21	11.2 ± 0.3
EZH2_shRNA_#2	12.1 ± 1.1
Irradiation + Anti-miR-21	18.5 ± 0.3
Irradiation + EZH2_shRNA_#2	21.9 ± 0.9
Irradiation + EZH2 cDNA	8.7±0.6
Anti-miR-21 + EZH2 cDNA	6.3±0.8
Irradiation + Anti-miR-21 + EZH2 cDNA	9.1±0.6

Supplementary Table 4: Effect of miR-21 or EZH2 on cell apoptosis of LCSCs after Cisplatin treatment ($\bar{x} \pm s$, n = 6)

Group	Apoptosis (%)
Control	2.9±0. 4
Cisplatin	14.1± 0.5
Anti-miR-21	11.2 ± 0.3
EZH2_shRNA_#2	12.1 ± 1.1
Cisplatin + Anti-miR-21	21.3% ± 0.2
Cisplatin + EZH2_shRNA_#2	22.2 ± 0.4
Cisplatin + EZH2 cDNA	9.2±1.2
Anti-miR-21 + EZH2 cDNA	6.9±1.3
Cisplatin + Anti-miR-21 + EZH2 cDNA	7.9±0.7
